# Resilience Training Programs in Organizational Contexts: A Scoping Review

**DOI:** 10.3389/fpsyg.2021.733036

**Published:** 2021-10-14

**Authors:** Ianina Scheuch, Natalie Peters, Max S. Lohner, Caroline Muss, Carmela Aprea, Bärbel Fürstenau

**Affiliations:** ^1^Faculty of Business and Economics, Chair of Business Education and Management Training, TU Dresden, Dresden, Germany; ^2^Business School, Chair of Business and Economic Education - Instructional Systems Design and Evaluation, University of Mannheim, Mannheim, Germany

**Keywords:** resilience, resilience training, resilience training programs, well-being, organizational context, scoping review

## Abstract

The importance of resilience for employees' well-being and performance at work has grown steadily in recent years. This development has become even more pronounced through the recent COVID-19 pandemic and its consequences, including major changes in occupational settings. Although there is increasing interest in resilience in general and a growing number of publications focusing on the development of resilience in particular, many questions remain about resilience training, especially in organizational contexts. The purpose of this scoping review is to uncover what is known about resilience training in organizational contexts. A systematic search of four databases for articles published through 2021 was conducted. A total of 48 studies focusing on resilience training programs in organizational contexts were included in this review. The review provides relevant insights into resilience training programs by focusing on program characteristics, target group, study design, and outcomes. Based on the results, the main aspects that concern the development of resilience training programs for organizational settings and requirements for the study design for empirical investigation were summarized. The results of the review highlight possible directions for future research and offer useful insights for resilience-enhancing training programs in organizations.

## Introduction

In times of ongoing global change and amid a trend of work intensification, today's employees face increased pressure at work, ranging from small to more chronic stressors, such as excessive job demands or challenging working conditions across different occupational contexts. In light of those challenges, adverse situations may not only affect employees' performance but can also seriously threaten their mental health and well-being (e.g., Schaufeli and Greenglass, 2001). This development has become even more pronounced during the recent COVID-19 pandemic and its consequences, including major changes in occupational settings (Teng-Calleja et al., 2020). Due to the need for social distancing, mandatory lockdowns, and isolation periods, the pandemic has brought with it even more challenges associated with work-related stress (Giorgi et al., 2020) and substantial costs to individuals and organizations. In response to those challenges, growing attention has been paid to resilience, which can be defined as an employee's ability to manage and positively overcome stress and adversity at work but also to grow though them (Mancini and Bonanno, 2009; King and Rothstein, 2010; Cooper et al., 2013; Fletcher and Sarkar, 2013; Johnston et al., 2015; Kossek and Perrigino, 2016). Therefore, the question arises regarding how to design effective resilience training programs that could help individual members of organizations improve their resilience and well-being.

Given the increased demands in organizational contexts and the importance of resilience, a growing number of resilience training programs have provided insights into training outcomes and elements. Studies including resilience training programs have been reported to show positive impacts on the mental health and subjective well-being of employees (e.g., Grant et al., 2009; Pipe et al., 2012). Moreover, some studies have also reported positive changes in performance or other work-related benefits (e.g., Grant et al., 2009; Pipe et al., 2012). A review of studies regarding resilience training in organizational contexts conducted by Robertson et al. (2015) and meta-analyses performed by Leppin et al. (2014) and Vanhove et al. (2016) all revealed support for the assumption that resilience training could positively affect employees' resilience as well as their well-being and performance at work. The reviews, however, also highlight that resilience training programs differ in their approaches and implementation and that “no single accepted theoretical framework or consensus statement exists to guide the development or application of those programs” (Leppin et al., 2014; p. 2). The purpose of this scoping review is to uncover what is known about resilience training programs in organizational contexts. By applying a scoping approach, we were able to build upon earlier reviews and summarize the state of research by integrating new insights from current studies published through 2021. In this article, we first present the applied review and analysis methods, followed by the results of our review. Finally, we discuss our findings and provide possible directions for future research.

## Methods

A literature review was conducted based on the guidelines for scoping reviews (Arksey and O'Malley, 2005, PRISMA-ScR: Tricco et al., 2018). According to Arksey and O'Malley's (2005) methodological approach for scoping reviews, the following five stages were conducted: (1) identifying the research questions, (2) identifying the studies, (3) selecting the studies, (4) extracting and charting the data, and (5) summarizing the results.

### Review Questions

This scoping review was conducted to address the following questions:

1. What training programs exist to improve the resilience of employees in organizational contexts?
2. What are the target groups of resilience training programs in organizational contexts?
3. Which concepts do these programs use and on which theories are they founded? What are their aims and content?
4. What are the (main) characteristics of resilience training programs in organizations?
5. Which methods and approaches are used to evaluate resilience training in organizations?
6. What are the (main) outcomes of resilience training programs in organizations?

### Identification of Studies

A systematic search was conducted between November 2020 and March 2021. We selected four databases—PubMed, PsycINFO, Business Source Complete (provided by EBSCO), and Web of Science. For each database, we developed an adequate research string that combined the term “resilien^*^” with “train^*^” or “intervent^*^” or “program^*^” or “promote^*^” and “work^*^” or “organi^*^ation” or “employ^*^” and searched within titles, abstracts, and keywords. Search limiters used included (when available): journal articles, English language, and abstract available. In addition to the search performed in the online databases, an additional search was performed through snowballing the reference lists of existing reviews and the publications identified in the database search.

### Study Selection

**Figure 1:**
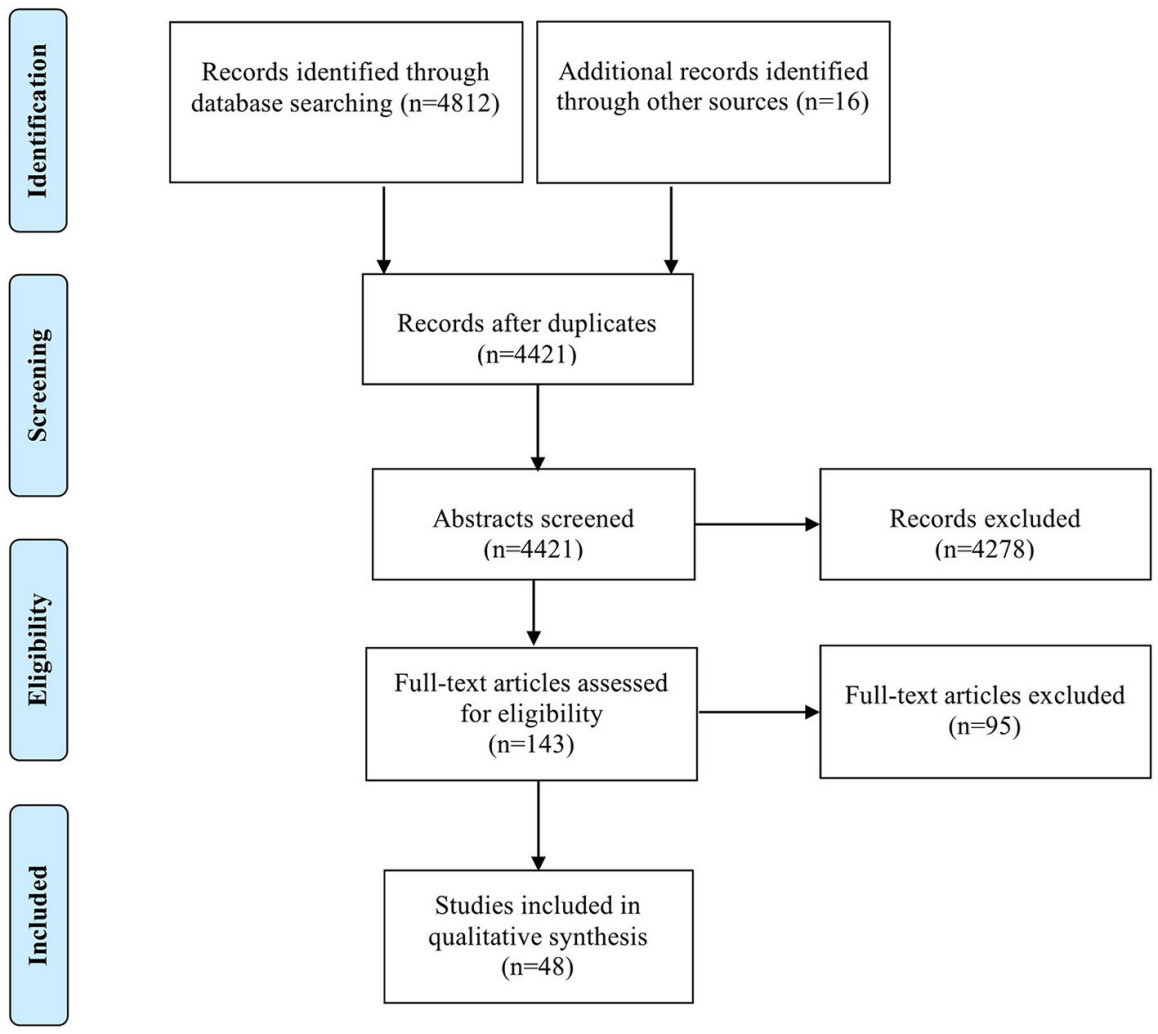
Flow diagram of articles identified and excluded according to PRISMA guidelines.

In a first step, the titles, keywords, and abstracts of the identified articles were screened. In a second step, four authors independently judged the relevance of the full-text articles and fine-screened the remaining articles using the selection criteria mentioned below, regarding participants, training characteristics, outcomes measures, and study design. To ensure rigor and high quality, the literature selection was documented using both inclusion and exclusion criteria. If judgements of single articles were inconsistent, the authors discussed their disagreements and achieved a consensus. Figure 1 depicts the stages of the paper-selection process.

For the purposes of this review, selection criteria were used according to a previous review by Robertson et al. (2015):

#### Participants

Any working (employee) sample (i.e., adults >18 years old). As we aim at a comprehensive understanding of work-related resilience training, we did not exclude any specific occupational context.

#### Training Characteristics

Any specifically resilience-based training, irrespective of content, duration, setting, or delivery media. In this sense, a training was classified as a resilience training if the study's authors labeled it explicitly as such or used similar wording or if the training increased resilience according to the study results.

#### Outcome Measures

These include resilience (as measured with specific resilience scales) as well-closely related constructs, such as individual mental health and well-being. Further outcomes include physical health, psychological functioning, and work performance (if applicable).

#### Study Design

All study designs were included (e.g., randomized-controlled trial, controlled trial, trial).

### Data Extraction

An Excel spreadsheet was used to maintain a systematic data-extraction and-analysis process. In the first stage, the relevant information from each study, including authors, year, study design, sample, and outcomes, were extracted. This step provided a general overview of aspects of resilience training programs found within the literature and a basis for a more detailed analysis. In the second stage of the review process, the results were synthesized in a narrative and tabular form by describing the resilience training programs and their characteristics and the outcomes. We organized and clustered the relevant results into themes, examining those that related to the research questions. To characterize the studies included in our review, we used five categories:

1. general overview (i.e., date of publication, occupational context, and country),
2. target group,
3. program characteristics: (i) program name, conceptual and/or theoretical background, aim, content; (ii) delivery mode; (iii) duration;
4. study description (i.e., design, data gathering, outcome measures, data analysis), and
5. outcomes.

## Results

### General Overview

We identified 48 relevant articles with a focus on resilience training programs in organizational contexts (see Table 1). Recently, there has been a steady increase in the number of resilience training publications, with more than half of the studies in this area having been published after 2017. The reviewed studies were conducted in different countries and occupational settings. Half of the studies were conducted in the United States, followed by Australia and other countries (the United Kingdom, Canada, and others). Most of the studies were conducted in health-care and high-risk occupational contexts (e.g., military, police, firefighters), followed by a smaller number of studies in public administration, business, or educational contexts.

**Table 1:** Resilience training programs.

**Main category**	**Reference**	**Program name**	**Delivery mode**	**Duration**
Multimodal	Babanataj et al. (2019)	Resilience training	live (f2f)	5 × 90–120 min
	Grant et al. (2009)	Multimodal intervention	live (f2f)	8–10 weeks
	Henshall et al. (2020)	Taking care of yourself to take care of others	live (f2f)	6 days over 12 weeks
	Kinman and Grant (2017)	Multimodal intervention	live (f2f)	3 days over 2 months
	Mahaffey et al. (2021)	Disaster worker resiliency training program (DWRT)	live (f2f)	4 hr
	Mealer et al. (2014)	Multimodal intervention	live (f2f)	12 weeks
	Mistretta et al. (2018)	Mindfulness-based resilience training (MBRT)Smartphone delivered resiliency-based intervention	mixed	MBRT: 6 weeks (120 min/week)Smartphone: 6 weeks
	Rogerson et al. (2016)	Workplace resilience program	live (f2f)	5 weeks (1 hr/week)
	van Agteren et al. (2018)	Resilience training	live (f2f)	2 days
SMART	Chesak et al. (2015)	Brief Stress management and resiliency training (SMART)	live (f2f)	1 × 90 min + optional 1 × 1 hr follow-up after 4 weeks
	Sharma et al. (2014)	Stress management and resiliency training (SMART)	distance	12 weeks
	Sood et al. (2011)	Stress management and resilience training (SMART)	live (f2f)	90 min + optional 30–60 min follow-up
	Sood et al. (2014)	Stress management and resilience training (SMART)	live (f2f)	90 min + 2 follow-up phone calls
	Werneburg et al. (2018)	Stress management and resiliency training program (SMART)	live (f2f)	12 weeks
Mindfulness	Aikens et al. (2014)	Online mindfulness intervention (modified MBSR)	online	7 weeks (1 hr/week)
	Crowder and Sears (2017)	Mindfulness-based stress reduction (MBSR)	live (f2f)	8 weeks (2.5 hr/week) + 1 full-day weekend session
	Fortney et al. (2013)	Abbreviated MBSR program (MBSR)	live (f2f)	18 hr + 2 × 2 hr follow-up
	Christopher et al. (2018)	Mindfulness-based resilience training (MBRT)	live (f2f)	8 weeks (2 hr/week) + extended 6 hr class in 7th week
	Rees et al. (2020)	Mindful self-care and resiliency program (MSCR)	mixed	4 hr (f2f) + 3 × 1 hr video-conference follow-up sessions
	Jennings et al. (2013)	Cultivating awareness and resilience in education (CARE)	live (f2f)	4 days over 4–6 weeks + intersession phone coaching + booster 2 months after
PAR	Foster et al. (2018)	Promoting adult resilience program (PAR)	live (f2f)	2 days (3 weeks apart)
	Liossis et al. (2009)	Promoting adult resilience program (PAR)	live (f2f)	7 weeks (11 sessions × 60 min)
	Millear et al. (2008)	Promoting adult resilience (PAR)	live (f2f)	11 weeks (1 hr/week)
R2MR	Carleton et al. (2018)	Road to mental readiness (R2MR)	live (f2f)	1 single session (no time specified)
	Dobson et al. (2020)	Anti-stigma workplace intervention “working mind” (adaption of R2MR)	live (f2f)	*not specified*
	Fikretoglu et al. (2019)	Road to mental readiness (R2MR)	live (f2f)	160 min
RAW	Joyce et al. (2018)	Resilience@work mindfulness program (RAW)	online	self-paced intervention (6 sessions à 20–25 min)
	Joyce et al. (2019)	Resilience@work mindfulness program (RAW)	online	self-paced intervention (6 sessions à 20–25 min)
Coaching	Dyrbye et al. (2019)	Professional coaching intervention	distance (telephone)	5 months (1 × 1 hr coaching session + 5 × 30 min sessions every 2-3 weeks)
	Sherlock-Storey et al. (2013)	Brief coaching for resilience	live (f2f)	3 × 90 min over 6 weeks
Others	Abbott et al. (2009)	Resilience online (ROL)	online	10 weeks
	Agarwal et al. (2020)	Sustaining resilience at work (StRaW)	live (f2f)	2 days
	Arble et al. (2017)	Imagery-based trauma prevention training program	live (f2f)	5 × 90 min (on consecutive days)
	Arnetz et al. (2009)	Police trauma resilience training	live (f2f)	10 weeks (2 hr/week) + 1 initial session
	Buchanan and Reilly (2019)	Heart math resiliency training	live (f2f)	8 hr monthly class
	Burton et al. (2010)	Psychosocial resilience training (READY program)	live (f2f)	11 × 2 hr over 13 weeks
	Carr et al. (2013)	Master resilience trainer	live (f2f)	12 weeks (weekly sessions)
	de Visser et al. (2016)	Stress resilience training system (SRTS)	online (app-based)	not specified
	Grabbe et al. (2020)	Community resiliency model class	live (f2f)	3 hr
	Heather et al. (2019)	LAMDU resilience program	mixed	*not specified*
	Kim et al. (2018)	Mobile video conference-based Intervention (SMART-3RP)	online (app-based)	4 weeks (1 hr/week)
	McCraty and Atkinson (2012)	Coherence advantage stress resilience and performance enhancement	live (f2f)	3 × 4 hr over 1 month
	Pehlivan and Güner (2020)	Compassion fatigue resiliency program	live (f2f)	Short-term: 5 hr for 2 days; long-term: 5 weeks (2 hr/week)
	Pidgeon et al. (2013)	Mindfulness with metta training program (MMTP)	live (f2f)	2 1/2 days + booster sessions at 1 and 4 months
	Pipe et al. (2012)	Transforming stress	live (f2f)	1 × 5 hr + 1 × 2 hr
	Tonkin et al. (2018)	Well-being intervention (well-being game)	online (app-based)	1 month
	Waite and Richardson (2003)	Personal resilience and resilient relationships (PRRR)	live (f2f)	5 weeks (7 hr/week) + follow-up review sessions
	Weber et al. (2019)	Mobile health intervention (Kelaa mental resilience)	online (app-based)	4 weeks (6–7 daily sessions à 2–4 min, max. 28 sessions)

### Target Group

The target groups comprised participants from different occupational contexts, with the most training programs (nearly 40%) addressing employees working in health care. These training participants included, e.g., nurses, residents, and physicians, coming from various disciplines and representing different hierarchical levels of the organization.

Employees working in health administration and health management represented the target group in six studies (Grant et al., 2009; Pipe et al., 2012; Sharma et al., 2014; van Agteren et al., 2018; Buchanan and Reilly, 2019; Heather et al., 2019). In contrast to frontline employees in health care, their work in administration does not include direct medical contact with patients. Four of these six studies, however—those by Buchanan and Reilly (2019), Pipe et al. (2012); Sharma et al. (2014), and van Agteren et al. (2018)—additionally included health-care employees in their target groups.

Eleven studies investigate employees working in high-risk environments that involve ensuring public safety and security, such as members of military services (Carr et al., 2013; de Visser et al., 2016; Fikretoglu et al., 2019), police officers (Arnetz et al., 2009; McCraty and Atkinson, 2012; Arble et al., 2017; Carleton et al., 2018; Christopher et al., 2018), firefighters (Joyce et al., 2018; 2019), and disaster workers (Mahaffey et al., 2021).

Nine studies were conducted in the occupational context of business management or public administration (Waite and Richardson, 2003; Abbott et al., 2009; Liossis et al., 2009; Burton et al., 2010; Sherlock-Storey et al., 2013; Rogerson et al., 2016; Tonkin et al., 2018; Agarwal et al., 2020; Dobson et al., 2020). The corresponding target group consisted of employees working in public and private corporations and in different business units, such as sales, tax, accounting, or human resources.

In three studies (Pidgeon et al., 2013; Crowder and Sears, 2017; Kinman and Grant, 2017), the target group consisted of employees working in social care: for instance, as social workers. One study was directed at teachers (Jennings et al., 2013).

The remaining studies (Millear et al., 2008; Aikens et al., 2014; Kim et al., 2018; Weber et al., 2019) did not clearly specify their target groups regarding the occupational background of study participants. Instead, they identified their target groups simply as employees. These employees worked in a multinational chemical corporation (Aikens et al., 2014), a resource sector company (Millear et al., 2008), in different European businesses in Germany, England, and Northern Ireland (Weber et al., 2019), or were characterized as full-time employees. As the studies did not outline the employees' occupations, however, they cannot be assigned to a specific occupational context.

### Program Characteristics

#### Program Name, Background, Aim, Content

All included studies referred to at least one specific category of training programs, resulting in a total of eight different categories of resilience training programs (see Table 1). Nine studies focused on *multi-modal resilience programs*, which differed in name, were based on more than one conceptual and/or theoretical background and applied multifaceted contents. The aims of these multi-modal training programs were quite heterogeneous. Many focused on improving resilience (Grant et al., 2009; Mealer et al., 2014; Rogerson et al., 2016; Kinman and Grant, 2017; Babanataj et al., 2019; Mahaffey et al., 2021). While three aimed to decrease stress, another three studies focused on improving well-being or mental health through the training programs. While previous reviews (Robertson et al., 2015; Vanhove et al., 2016) classified programs with different cognitive-behavioral techniques as *multi-modal*, this review used and broadened the category to include programs that employ multiple theoretical/conceptual approaches and contents. Part of these theoretical foundations was positive psychology, as well as cognitive (-behavioral) and mindfulness approaches. The various contents of the training programs include, for example, relaxation training, goal-setting, problem-solving, meditation, coaching, feedback, psycho-education on resilience, and reflective and critical thinking.

Five studies applied the *Stress Management and Resilience Training (SMART)* program (Sood et al., 2011, 2014; Sharma et al., 2014; Chesak et al., 2015; Werneburg et al., 2018). All studies focused on increasing resilience, and two also focused on improving mindfulness (Sharma et al., 2014; Chesak et al., 2015). Decreasing stress and/or anxiety was an aim of four out of five studies. One study also focused on improving quality of life (Sood et al., 2014). This training program itself is based on Attention and Interpretation Therapy (AIT), which teaches learners to focus their attention on the present moment and to defer unrefined judgments. Learners are also taught to cultivate and guide their interpretations by higher-order principles such as forgiveness, acceptance, gratitude, compassion, and life's meaning, instead of superficial prejudices, (Sharma et al., 2014; p. 248).

Three studies applied the Mindfulness-Based Stress Reduction (MBSR) program, which aimed at promoting well-being and positive organizational behavior, resilience or job satisfaction, quality of life, or compassion. Reducing burnout was also an aim of one study. The *Mindfulness-Based Resilience Training (MBRT)* and *Mindful Self-Care and Resiliency (MSCR)* were each used by one other study. While the first targeted stressors inherent to police work, the other aimed to increase well-being. The term “mindfulness” can be defined as “paying attention in a particular way: on purpose, in the present moment, and non-judgmentally” (Kabat-Zinn, 1994; p. 4) and has its roots in Buddhist philosophy. Mindfulness, as a factor in improving health-related aspects like well-being and stress, was part of six studies in this review (Fortney et al., 2013; Jennings et al., 2013; Aikens et al., 2014; Crowder and Sears, 2017; Christopher et al., 2018; Rees et al., 2020). The specific approach to mindfulness differed among the authors. *MBSR*-founded by Kabat-Zinn at the University of Massachusetts Medical Center-is a “well-researched and clinically useful program widely recognized as a healthy way to manage symptoms of stress” (Fortney et al., 2013; p. 413). It includes various mindful exercises, such as body-scanning, yoga, or walking meditation. *MBRT*, on the other hand, integrates *MBSR* and Acceptance-Commitment Therapy (ACT). It “incorporates two practices: learning mindfulness skills to deal effectively with unpleasant/unwanted thoughts or experiences; and learning resilience skills to foster positive growth and behavior in keeping with one's intentions and values” (Mistretta et al., 2018; p. 560). The *Mindful Self-Care and Resiliency (MSCR)* program includes themes of “introduction to mindfulness, staying present, allowing/letting be, thoughts as thoughts, and review and planning for the future” (Craigie et al., 2016; p. 767). The *Cultivating Awareness and Resilience in Education (CARE)* program developed by Jennings et al. (2013), on the other hand, combines mindfulness and compassion practices and aims to reduce stress and improve performance.

The *Promoting Adult Resilience (PAR)* program was applied in three other studies (Millear et al., 2008; Liossis et al., 2009; Foster et al., 2018). Its aim is to promote resilience, mental health, and well-being (in the working population). This training program comprises seven main topics: (1) understanding personal strengths and resilience, (2) understanding and managing stress, (3) challenging and changing negative self-talk, (4) practicing changing negative self-talk, (5) promoting positive relationships, (6) problem-solving and managing conflict, and (7) “bringing it together.”

The *Road to Mental Readiness Program (R2MR)* program was applied in three studies (Carleton et al., 2018; Fikretoglu et al., 2019; Dobson et al., 2020). One study aimed to increase mental health literacy and stress-management skills, another to improve short-term performance and long-term mental health, and the third to improve resilience and reduce stigmata. The training program focuses on teaching four major skills (the “Big 4”) to the participants: tactical breathing, goal-setting, visualization, and self-talk (Fikretoglu et al., 2019).

Two studies reported on the *Resilience@Work Mindfulness Program* (*RAW*; Joyce et al., 2018, 2019). The aim of this training program (and in both studies) was to enhance psychological resilience in high-risk workers (Joyce et al., 2018, 2019). The training program involves “mindfulness training, psycho-education, and a range of skills and strategies drawn from evidence-based therapies” (Joyce et al., 2018; p. 3). These other therapies are Acceptance-Commitment Therapy (ACT), Mindfulness-Based Stress Reduction (MBSR), and Compassion-Focused Therapy.

Two studies applied a *professional coaching program* and aimed to improve either resilience-related behaviors (e.g., making use of a support network) or enhance well-being, job satisfaction, resilience, and fulfillment in physicians and a measurable reduction in burnout (Dyrbye et al., 2019). The specific content was individualized regarding the needs of the respective coachee. In one training, this was analyzed through an initial coaching session about needs, values, goals, and forming a relationship with the coachee. The subsequent sessions followed a structure: (1) check-in, debrief on the strategic action the participant has taken since the last session, manage the progress, and review accountability; (2) plan and set goals; (3) design actions to incorporate into daily life; (4) commit to the next step; and (5) check out and summarize. The other coaching program was briefer and included three sessions that focused on psycho-education about resilience areas and supporting goal-setting regarding resilience and well-being. A short review of the coaching progress and future goal-setting beyond the coaching program was also part of the coaching sessions.

Of the 48 studies analyzed, the remaining 18 studies focused on *various other training programs*. Similar to the multi-modal training programs, these included not only mixed and heterogeneous content but also various aims, such as promoting well-being, reducing stress, or enhancing resilience or resilience-related concepts (e.g., self-efficacy, hope). Four of these studies shared the conceptual basis of self-regulation toward stress responses via technology (McCraty and Atkinson, 2012; Pipe et al., 2012; de Visser et al., 2016; Buchanan and Reilly, 2019) but applied different theoretical backgrounds (e.g., theory of human caring, physiological coherence). Two articles were based on the Penn Resiliency Program (PRP), while the other twelve applied concepts and/or backgrounds that ranged from self-determination theory (Tonkin et al., 2018) to compassion fatigue (Pehlivan and Güner, 2020), imagery-based emotional exposure (Arble et al., 2017), and cognitive therapy or peer support systems approaches. The content also varied among these specific training programs, often including psycho-education on health-related subjects (e.g., stress, resilience, sleep science, compassion fatigue).

#### Delivery Mode

In terms of delivery mode of the resilience training programs, the characteristics, delivery media, form of delivery, and form of interaction of these training programs were analyzed. Thirty-five programs were delivered on a face-to-face basis. Seventeen of these were held in groups: twelve included group work and exercises alone (e.g., as homework), and three included one-on-one sessions or sole participation with support (e.g., through a coach). Three face-to-face training programs did not specify the interaction (Carr et al., 2013; Carleton et al., 2018; Pehlivan and Güner, 2020).

In eight studies, an online training was conducted, four of which were app-based (de Visser et al., 2016; Kim et al., 2018; Tonkin et al., 2018; Weber et al., 2019). Two training programs included mixed delivery media through online and face-to-face sessions (Heather et al., 2019; Rees et al., 2020). Of these ten training programs, seven were conducted on a one-on-one basis with one participant and trainer or implemented for solo participation with or without support (e.g., through a virtual partner). Two of these online and mixed training programs included group interaction, and one training did not specify how the participant(s) and potential trainers interacted.

A study by Mistretta et al. (2018) included two training programs. One delivered the intervention face-to-face, while the other applied smartphones as delivery media. While the first one was held in a group, the second was designed for sole participation without further outside support. Another single training applied its training via telephone (Dyrbye et al., 2019); therefore, it was based on a one-on-one interaction. One study used neither a digital nor a face-to-face delivery format, as the sessions were self-directed via analog-written materials as delivery media (Sharma et al., 2014). Here, participants handled the tasks alone, without any further support.

#### Duration

Of the ten online and mixed (face-to-face and online) training programs, two featured a self-paced training, one without a time frame and the other with a time frame of 3.5–6 weeks. Five training programs were conducted in a time span between 4 and 8 weeks (with varying daily or weekly sessions), and two gave no specific information about their duration. The training with self-directed learning through written material employed a 12-week duration (Weber et al., 2019), and the one held by telephone included six sessions over a span of 5 months (Dyrbye et al., 2019).

The 35 face-to-face training programs ranged from a single training session (e.g., Sood et al., 2011; Chesak et al., 2015; Carleton et al., 2018) to 11 sessions over 13-week period (Burton et al., 2010). The length of each session also varied from 60 minutes (e.g., Millear et al., 2008; Rogerson et al., 2016) up to 6 full-day sessions (Henshall et al., 2020). One of the face-to-face interventions gave no further insight into its duration (Dobson et al., 2020). Some studies only gave an overview of the length of their overall training program (e.g., Grant et al., 2009; Carr et al., 2013; Agarwal et al., 2020), such as “2-day course,” “8–10-week period,” or “12-week period with weekly sessions.”

### Study Description

#### Study Samples and Groups

Of the 48 articles included in this review, 23 were based on randomized controlled trials (RCTs), five used a controlled trial (CT), one study used a cluster RCT, and 19 ran a trial (T) with no control group. The included studies' sample sizes ranged between 9 (Agarwal et al., 2020) and 2,202 (Fikretoglu et al., 2019) participants, with a median of 49 participants (*M* = 115.76*, SD* = 321.46). Sample sizes of more than 100 participants were reported in 10 articles.

#### Design, Data Gathering, Outcome Measures, Data Analysis

Two studies reported a single measurement point after the training (Heather et al., 2019; Agarwal et al., 2020). The remaining 46 studies used at least one measurement point before and one after the training. Seventeen of these studies implemented a pre-test and an immediate post-test with measurements directly before and after the training. In seven studies, pre-tests and delayed post-tests were used with one measurement directly before the training and a second measurement between 1 month and 1 year after the end of the training (*med* = 3 months, *M* = 4.42 months, *SD* = 3.54 months). The remaining 22 studies applied pre-, post-, and follow-up tests. Follow-up measurements were collected between 2 weeks and 1 year after the end of the training. As it was used in eight of the 22 studies, a delay of 3 months between the end of the training and the follow-up measurement was the most frequently used timespan, as well as the median (*M* = 4.48 months, *SD* = 3.23 months). Five of the studies using a pre-, post-, and follow-up test used multiple follow-up measurement points. Finally, the study by Weber et al. (2019) used a pre-, post-, and follow-up test, and also included a measurement at the mid-point of the training program.

Forty-seven studies included a quantitative evaluation of the training outcomes, while one study (Agarwal et al., 2020) focused on a qualitative outcome evaluation via interviews. Quantitative outcomes were gathered mainly through self-report questionnaires, but some featured performance tests (Fikretoglu et al., 2019), physiological measures like heart rate and blood pressure (Arnetz et al., 2009; McCraty and Atkinson, 2012), observer assessments (Arnetz et al., 2009; de Visser et al., 2016), and organizational performance data (Abbott et al., 2009) to evaluate the training outcomes.

The CD-RISC (Connor and Davidson, 2003) was the most frequently used measure in the reviewed studies; it was used in 16 of them. In the studies using this scale, six included the 10-item short form, and 10 studies used the 25-item version. Other frequently used self-report scales were the Perceived Stress Scale (Cohen et al., 1983), included in 13 studies, the Depression and Anxiety Stress Scale 21 (Lovibond and Lovibond, 1995), included in ten studies, and the Maslach Burnout Inventory (Maslach et al., 1986), included in five studies. All remaining measures occurred in fewer than five reviewed articles.

Common variables can, however, be identified in the outcome measures, with scales for resilience and coping included in 37 studies, scales for stress included in 30 studies, scales for mental health disorders included in 27 studies, and scales for well-being and quality-of-life outcomes included in 18 studies. Additionally, 13 studies included outcomes for training satisfaction or feasibility, which were mostly gathered at the end of the training and often included open-answer instruments or self-developed scales.

Twenty-nine of the 47 studies that included a quantitative measurement featured a scale directly related to the occupational setting or work context: e.g., Resilience at Work (Rogerson et al., 2016), Teachers' Sense of Efficacy Questionnaire (Jennings et al., 2013), and Police Stress Questionnaire (Christopher et al., 2018). Scales for general characteristics and traits not bound to a work-related context are included in 46 articles.

For statistical analysis of the training effectiveness, *t*-tests were the most commonly used method, being present in 24 studies. Eight of those studies include *t*-tests in conjunction with other analysis methods (e.g., as post-*hoc* analyses following an ANOVA), while the remaining 16 articles used *t*-tests as their sole analysis method. Analysis of variance or covariance was performed in 16 studies, and regression models in 10 studies. Effect sizes were reported in 22 studies, with frequent use of Cohen's *d* in 13 studies.

### Outcomes

Of the 27 studies, 17 found a significant positive effect of the training on the resilience variables. The reported effects ranged from small to large effect sizes. Similar results were reported for outcomes regarding stress: 16 of the 25 studies that reported significance show a significant decrease in this variable, with the reported effect sizes ranging from small to large. Regarding measures for psychological disorders, 14 of the 26 studies calculating significances for these outcomes found significant changes in at least one of the mental-health outcomes (e.g., depression, anxiety, PTSD, burnout). Effect sizes ranged from no effect to large effects, but large effects were only found for the reduction of anxiety in the studies by Sood et al. (2011, 2014) and of negative mood in the study by Arnetz et al. (2009). Of the 18 studies that included measures for well-being or quality of life, all authors reported *p*-values, but only ten articles reported results lower than *p* = 0.05 threshold. Effect sizes ranged from no effect to large effects, with large effects occurring in the study by Sood et al. (2011, 2014) for quality of life and in the study by Mistretta et al. (2018) for well-being.

Twelve of the 20 studies that reported significance for occupational or work-related scales found a significant effect on at least one of these outcomes. Effect sizes ranged from no effect to large effects, with large effects for resilience at work in the study by Rogerson et al. (2016), work family spillover in the study by Liossis et al. (2009), and observer performance rating in the study by Arble et al. (2017).

Few studies statistically tested participants' satisfaction with and feasibility of the training, but the reported findings showed that the training programs were positively evaluated by most participants. Several studies, however, reported a high dropout rate over the course of the intervention.

## Discussion

First, the reviewed studies showed that resilience training programs are usually applied in high-risk contexts and focused on employees who are regularly confronted with high levels of stress (e.g., police officers, military members, or doctors). Even though the prevention and treatment of stress and burnout, as well as the promotion of well-being and resilience, are particularly important in these contexts, studies of employees in regular business settings (e.g., office work) remain rare. Nevertheless, we already know that these employees also face increasing pressure at work (e.g., Fletcher and Sarkar, 2013). Furthermore, as resilience has been conceptualized as a context-related construct (Kossek and Perrigino, 2016), it cannot be assumed that one resilience training is also relevant and can be conducted without adaptation in another context. Moreover, further attention should be paid to employees in managerial posts. Here, issues like standard overtime, work–life imbalance, or an irregular schedule are often cause for burnout or early retirement (Foerster and Duchek, 2017). Although there is recent research concerning organizational leaders' resilience and leaders' resilience-enhancing factors (e.g., Bossmann et al., 2016; Foerster and Duchek, 2017), no specific training programs have yet been developed and evaluated.

Second, a broad range of different training programs have been applied in organizations. Program characteristics, such as conceptual backgrounds, aims and contents, vary widely. As this heterogeneity has been stated by other reviews before (e.g., Robertson et al., 2015; Vanhove et al., 2016), our approach included clustering the articles due to the applied training programs (e.g., RAW, R2MR) or their common basis, such as mindfulness. Building on our results, multi-modal and specific single-focused training programs can be distinguished. A lot of studies focused on multi-modal resilience programs, which were based on more than one conceptual and/or methodological background and vary in their aims and contents. These contents include aspects, such as psycho-education about various health-related topics, relaxation techniques, social support, reflective thinking, goal setting, and/or problem solving. As mentioned before, some studies applied a single-focused (e.g., professional coaching or mindfulness) program or focus on other specific resilience training programs (e.g., Imagery-Based Trauma Prevention Training Program). Taken together, even though the number of studies on resilience development in the organizational context has risen largely, research is still in an early developmental stage.

Third, in terms of delivery mode (i.e., delivery media and forms of interaction) of the resilience training programs, the reviewed studies show a range of different strategies. While most of the studies focused on training programs applied on a face-to-face basis, several implemented online training, and a few applied approaches that combined online and face-to-face sessions. In keeping with increasing digitalization and—over the past 17 months—current regulations designed to contain the spread of COVID-19, numerous online-based training programs have been developed in recent years. Their effectiveness, however, remains to be examined, especially as Vanhove et al. (2016) meta-analysis showed computer-based training programs to be less effective than face-to-face programs. Additionally, the form of interaction should be taken into account. Most training programs applied forms of group interaction or a combined form of group and individual work (e.g., completing worksheets at home). Some conducted one-on-one meetings between a participant and an instructor (e.g., a digital or live coaching session) or individual work without support. Most of the latter categories were part of online-based programs. Given the discussed aspects—that is, effectiveness of digital or face-to-face formats and interaction forms in training programs—blended learning could combine the strengths of both approaches (combination of online module and offline learning) and therefore be a flexible, cost-effective way to strengthen employees' resources (see, e.g., Tonkin et al., 2018). Additional research is needed to gain further insight into the feasibility and effectiveness of different delivery and interaction formats.

Forth, another important aspect that varied between the resilience training programs was their duration. Results show that training programs range from short, single-session ones to regular weekly and/or full-day sessions. Despite the documented effects of short-term training sessions (e.g., Sood et al., 2011), the application of what has been learned, must be repeatedly applied in practice and transferred to new or other tasks (e.g., Andergassen et al., 2014). This could be achieved through short-term refresher courses that can become flexible, effective extensions of full training programs. These were applied in some training programs as “booster sessions,” at, for example, 1 and 4 months after the initial training (Pidgeon et al., 2013) or via follow-up sessions or phone calls (Sood et al., 2011; Sharma et al., 2014; Chesak et al., 2015). Because this practice was applied in only a few of the studies, regular (e.g., weekly) and follow-up courses should generally be part of future implementations, instead of the previously applied one-time sessions. This will not only allow participants to overcome potential shortcomings in learning and applying new knowledge and skills, but will also allow study conductors to run long-term analysis.

Fifth, examining the study descriptions, among the studies reviewed, fewer than half of the studies evaluated resilience training programs in organizations following a RCT design. In addition, a high prevalence of studies lacked a control group, making it difficult to control for external effects and/or generalize findings. Future resilience training studies should include control groups and use the RCT design to produce more reliable findings. Ideally, control groups should be recruited from the same occupation and organization. The control group could either receive an alternative non-resilience related training or receive the same resilience training after the last measurement of the experimental group (waiting list control group). Additionally, suitable sample sizes should be calculated and procured to ensure the success of such designs. The effect sizes reported in the existing studies can form a basis for calculating sample sizes for future training evaluations of existing programs. When planning a training evaluation, this review also highlighted the importance of recruiting more than the minimum number of suitable participants due to high dropout rates in some programs (e.g., Carr et al., 2013; Buchanan and Reilly, 2019). The included studies offer no insight into why participants drop out of resilience training, and a scientific evaluation is needed to detect whether these reasons lay in the organizational context or the acceptance of participants. As authors have treated their report on drop-out rates very differently, no consistent picture emerges regarding the existence, number of, or reasons for drop-out.

Sixth, the included outcome measures to evaluate resilience training programs showed a large variety. This underlines the observations by Windle et al. (2011) that there is no “gold standard” in resilience measurements and shows that this observation can be confirmed for the studies included in this review. Few studies describe why the chosen resilience measures were used, but CD-RISC was the most often used scale to assess resilience. Resilience measured via CD-RISC represents trait resilience (e.g., Singh and Yu, 2010; Wollny and Jacobs, 2021), but in the training context resilience is operationalized as a changeable variable; and thus, it is important to use resilience scales that match the theoretical considerations (Linz et al., 2020). With most studies finding positive effects for resilience outcomes and a reduction in stress, resilience training programs seem to be effective and fulfilling their purpose. Effectiveness was also shown for mental-health outcomes and well-being, but fewer studies could report significant improvements in these variables. Work-related outcomes seem to be positively influenced by resilience training, especially if they are closely related to the concept of resilience (e.g., resilience at work, work–family spillover). While significant positive relationships with various desirable outcomes could be found, the studies allow no conclusions to be drawn about causality. The heterogeneous characteristics of the training programs did not allow to mark single training characteristics as advantageous or disadvantageous. It is only possible to acknowledge that most resilience training programs showed positive effects.

Finally, we can conclude that recent research has not yet systematically focused on instructional design aspects of resilience training programs. The description of the design and implementation of resilience training programs (e.g., specific learning objectives or assessment approaches) in some studies was limited, precluding a more in-depth analysis and presentation of the findings. Some aspects, like duration, delivery mode, forms of interaction, and an overview of the content, have been part of the training programs in the analyzed studies. However, these aspects do not seem to have been deliberately designed according to an instructional framework. Regarding the current results of this review, it can be seen that most of the authors have not included this aspect in their research or—at least—have not reported on it in their publications. This needs to be further analyzed, with special regard to instructional design of resilience training programs.

## Conclusion and Future Directions

In recent years, research has leveraged and extended the findings generated in previous reviews. Still, while progress has been made, some research gaps remain that should be addressed by future studies to provide a more comprehensive understanding of resilience training in organizational contexts. We hope that this scoping review can raise awareness of the importance of resilience training research in an organizational context. Specifically, future research should address the roles of different resilience training programs, their conceptual backgrounds, and their related outcomes more precisely to help organizations choose or develop resilience training programs in three ways. First, by taking a closer look at different contexts, it becomes apparent that the identified professional backgrounds are not equally represented in the considered studies. For example, most training programs are implemented with general employees or employees working in high-risk environments; only a few programs focus on employees in business settings. In addition, few studies specify the hierarchical positions of their participants, so there is less knowledge of the level of resilience development for specific target groups within organizations. Second, as we provided insights into program characteristics and outcome measures, future research could consider how different program characteristics or, especially, delivery modes (e.g., face-to-face vs. online) may affect the outcomes of resilience training programs. Overall, more research is needed to implement the criteria for success in the design and implementation of resilience training programs in organizations themselves as well as the training conditions of the organizational context. Third, an interesting future direction has opened up in the research regarding the instructional frameworks and designs of resilience training programs for employees. Although some design aspects were included in the examined training programs, these aspects do not seem to have been deliberately designed according to an instructional framework or include instructional approaches. Future studies could, therefore, focus on instructional design aspects and analyze which forms of learning are especially relevant for resilience training programs in general and in the organizational context in particular.

## Author Contributions

IS provided the idea for the study, designed the research plan and the review protocol, co-developed the assessment, supervised the analyses, and co-wrote the manuscript. NP co-developed the assessment, conducted the analyses and interpretation, and co-wrote the manuscript (program characteristics). ML co-developed the assessment, conducted the analyses and interpretation, and co-wrote the manuscript (study design and outcomes). CM co-developed the assessment, conducted the analyses and interpretation, and co-wrote the manuscript (target group). CA and BF supervised the analyses and were involved in preparing and reviewing the manuscript. All authors contributed to the article, reviewed the results and approved the final version of the manuscript.

## Conflict of Interest

The authors declare that the research was conducted in the absence of any commercial or financial relationships that could be construed as a potential conflict of interest.

## Publisher's Note

All claims expressed in this article are solely those of the authors and do not necessarily represent those of their affiliated organizations, or those of the publisher, the editors and the reviewers. Any product that may be evaluated in this article, or claim that may be made by its manufacturer, is not guaranteed or endorsed by the publisher.
